# Supplementary material for: New traps for the capture of Aedes aegypti (Linnaeus) and Aedes albopictus (Skuse) (Diptera: Culicidae) eggs and adults
Source: PLoS Negl Trop Dis. 2021 Apr 16;15(4):e0008813. doi: 10.1371/journal.pntd.0008813 (PMC8081340; doi:10.1371/journal.pntd.0008813)
Supplement: Attachment — Submitted filename: Comments.doc [file pntd.0008813.s001.doc]

To Dr. Claire Donald

*Associate Editor*

PLOS Neglected Tropical Diseases,

The manuscript "New traps for the capture of *Aedes aegypti* (Linnaeus) and *Aedes albopictus* (Skuse) (Diptera: Culicidae) eggs and adults" is relevant to public health and thus should be considered for publication. However, there are issues to be addressed, which I explain below in detail; in this regard, I strongly encourage the authors to make the attached corrections. The main concerns are about the short sampling period and the concentration of capture.

Thank you again for your consideration.

Best regards,

__________________________________________________________________

The manuscript "New traps for the capture of *Aedes aegypti* (Linnaeus) and *Aedes albopictus* (Skuse) (Diptera: Culicidae) eggs and adults" is relevant and is in line with the scope of the Journal. However, the following modifications need to be addressed to make it publishable:

*Comments*

Despite the authors' efforts to improve a technology that aims to monitor these species in an alternative and efficient way, I have concerns about it because existing traps perform this same functionality efficiently to achieve these same purposes. For example, concerning the cost of this monitoring tool: is it viable and feasible production to manufacture it on a large scale?

Will the authors add the patent registration number of this trap, or is it the object of another patent? It is important to be aware of the legislation of each country.

Abstract Line 33. What were the criteria to define 20 traps to test efficiency?

Line 132-141. These are coordinates of a single point of the area. Please provide the coordinates of at least four points at the boundaries of the studied area, as well as those of the three sampled areas separately.

Lines 142-152. I recommend that the authors make a more accurate description, in addition to referencing an image of the traps to facilitate the understanding of its functionality. It is important to emphasize that it is a new trap but characterizes a new adaptation of existing traps, the UELtrap-E and UELtrap-EA, which capture *A. aegypti* and *A. albopictus* eggs, respectively.

Line 152. It is not clear which entomological glue is that.

Lines 169-170. What was the criterion used to outline the distances between the collection areas?

Line 248. I believe that these results are precipitated, considering the average time of experimental field test sampling. You need to add data from the scientific literature to support this information in a seasonal period of both experimental areas.

Lines 375-381. Could the experimental seasonal period have directly influenced the collection results in both sample areas and generated a false, biased traps efficiency?

Line 433-439. This is not clear. Why not strengthen the use of traps inside homes, considering that *Aedes aegypti* is quite frequent inside the houses of urban and suburban areas (where the human population concentration is high)?
