# Supplementary material for: New traps for the capture of Aedes aegypti (Linnaeus) and Aedes albopictus (Skuse) (Diptera: Culicidae) eggs and adults
Source: PLoS Negl Trop Dis. 2021 Apr 16;15(4):e0008813. doi: 10.1371/journal.pntd.0008813 (PMC8081340; doi:10.1371/journal.pntd.0008813)
Supplement: Attachment — Submitted filename: PNTD-D-20-01718.docx [file pntd.0008813.s002.docx]

The present manuscript entitled “**New traps for the capture of *Aedes aegypti* (Linnaeus) and *Aedes albopictus* (Skuse) (Diptera: Culicidae) eggs and adults**” is well written and organized it provides *Aedes* surveillance new methods to monitor mosquitos populations in specific area. However, the manuscript contains some important issues that need to be addressed. My comments are found below.

The objective of the study is clear and statistical analysis used support the conclusion. However, in the Methods there are some important issues:

- In study area, the authors need to describe the climate conditional (precipitation, temperature), traps installed environmental conditions (vegetation cover, urban area etc), and others important landscape factors related with *Aedes* distribution present in the UTFPR and INPA *campus*. Also, the authors need to include the map with the studies areas and collets points. What were the criteria to choose the buildings to installed the traps?

-Trap characteristics: Please, include the photos of each traps (UELtrap-E, UELtrap-EA, CRtrap-E and CRtrap-EA) identifying all the elements. Are UELtrap-EA and CRtrap-E designed to collect gravid *Aedes* female? If yes, the information needs to be in the text. Mention the estimated cost of traps.

- Collection eggs and adults of *Aedes* under field conditions: Please inform if the traps were and how were cleaned every 7 days? Were attractant solutions replaced every 7 days? Were attractant solutions transported to entomological laboratory to identify immature *Aedes* (larvae and pupae)? How was the material transported to the laboratory? How many people were needed to monitor the traps (field and laboratory).

The results are clear and well organized
with graphics and tables. However there are some important issues:

The proposal of the manuscript is to evaluate the effectiveness of 4 traps for the collection and capture of urban mosquitoes of the genus *Aedes*. The analyzes were carried out for the aggregated data over time, however the authors could inform the collects points with high and low number of the eggs and adults (male and female) collected per species. What factors could be associated with this finding, even knowing that it is a short period of time (in the discussion).

- Inform how many males and females were caught by adult mosquito species and by trap.
