# Supplementary material for: New traps for the capture of Aedes aegypti (Linnaeus) and Aedes albopictus (Skuse) (Diptera: Culicidae) eggs and adults
Source: PLoS Negl Trop Dis. 2021 Apr 16;15(4):e0008813. doi: 10.1371/journal.pntd.0008813 (PMC8081340; doi:10.1371/journal.pntd.0008813)
Supplement: Attachment — Submitted filename: Letter comments.docx [file pntd.0008813.s003.docx]

João Zequi

State University of Londrina, Brazil

zequi@uel.br

To Dr. Claire Donald

Associate Editor

PLOS Neglected Tropical Diseases

January, 26, 2021

Dear Dr. Claire:

We thank the reviewers and the journal's consideration for our article entitled: “New traps for the capture of *Aedes aegypti* (Linnaeus) and *Aedes albopictus* (Skuse) (Diptera: Culicidae) eggs and adults

Traps for *Aedes aegypti* and *Aedes albopictus* ”.

The reviewers' considerations helped to improve the quality of the article. We authors of the article met virtually due to the pandemic and discussed all the suggestions sent. The team considered all suggestions.

Some sections were modified according to suggestions. The characterization of the areas in terms of climate and vegetation issues was added, including more details to facilitate the understanding of the study areas.

A map with collection points for each area was built and inserted in the article according to suggestions to facilitate the location and characterization of the study area and its geographic dimension with georeferencing.

The team inserted a board containing photos of the four different traps and complemented it with more details in the text to facilitate didactically the changes made to the standard trap, as suggested by the reviewers.

All mention of control related to the trap was removed as suggested by the reviewers. Graphics have been improved in image quality.

The authors previously requested an extension to meet all demands. Below is a response to the reviewers' suggestions, including the modifications made, as well as justifications always linked to other articles published in journals of impact in the area. We also attached the new figures on the Platform, the draft review and the final version of the revised article.

The authors are grateful for the attention of the journal and the reviewers who contributed significantly to improvements in the article.

We hope to have met the demands and we are available for whatever else needs improvement.

Thank you for your consideration!

Sincerely,

João Zequi

State University of Londrina, Brazil

[zequi@uel.br](mailto:zequi@uel.br)

**Reviewer #3:** The objective of the study is clear and statistical analysis used support the conclusion. However, in the Methods there are some important issues:

- In study area, the authors need to describe the climate conditional (precipitation, temperature), traps installed environmental conditions (vegetation cover, urban area etc), and others important landscape factors related with *Aedes* distribution present in the UTFPR and INPA *campus*. Also, the authors need to include the map with the studies areas and collets points. What were the criteria to choose the buildings to installed the traps?

**The description of the climatic conditions of the different sampling sites related to vegetation and urban structure with factors that favor Aedes was included in the article as requested. A map was made as requested and designated as Figure 1 containing geographical coordinates and aerial satellite view with the blocks where the traps were placed.**

**The new text was:**

**“**The study was conducted in localities situated in the states of Paraná and Amazonas, South and North Regions of Brazil. In Paraná, the traps were installed around five buildings located on the *Campus* (74,000 m²) of the Federal Technological University of Paraná (UTFPR), in Londrina city (University Restaurant - 23 ° 18'28.51 "S 51 ° 6'56.52" W; Block A - 23 ° 18'28.24 "S 51 ° 6'54.04" W; Block B - 23 ° 18'27.40 "S 51 ° 6'54.34" W; Block P - 23 ° 18'27.21 "S 51 ° 6'50.77 "W; Block K - 23 ° 18'26.01" S 51 ° 6'48.77 "W) (Fig.1)

In Amazonas, the traps were distributed at five points located at *Campus* I (255,736.49 m²) of the National Institute for Amazônia Research (INPA), in Manaus city (Point 1 - 3 ° 5'47 "S 59 ° 59'10" W; Point 2 - 3 ° 5'43 "S 59 ° 59'11" W; Point 3 - 3 ° 5'40 "S 59 ° 59'15" W; Point 4 - 3 ° 5'41 "S 59 ° 59'17 "W; Point 5 - 3 ° 5'42" S 59 ° 59'15 "W) (Fig.1).

Fig 1. Location of the study area, demonstrating the distribution of the sample sites in the North and South Regions of Brazil.

The climate of Londrina is humid subtropical, with an average annual temperature of around 22 °C, relative humidity of around 70%, having hot summer and rain in all seasons (annual average between 1400 and 1800 mm) [42]. The study area is situated in the urban perimeter of Londrina and is daily attended by a high number of students and employees, constituting an important area for investigation and monitoring of mosquito vectors of pathogens.

In Manaus, the climate is humid equatorial with an average annual temperature of 26 °C, and a relative humidity of around 80%, with an annual rainfall of around 2,300 mm. The region has two well-defined seasons: rainy (December to June) and dry (July to November), based on rainfall and river levels [43].

The study site is a very wooded urban environment with native forest, which favors *A. albopictus* [2]*.* As in Londrina, the *Campus* are full of students and employees, being part of the local community.

-Trap characteristics: Please, include the photos of each traps (UELtrap-E, UELtrap-EA, CRtrap-E and CRtrap-EA) identifying all the elements. Are UELtrap-EA and CRtrap-E designed to collect gravid *Aedes* female? If yes, the information needs to be in the text. Mention the estimated cost of traps.

**The authors included, as requested, a board with photos containing details of all the traps used. It is designated as Figure 2.**

**“**Fig 2. Traps for capture of *Aedes* eggs and adults under field conditions: A) UELtrap-E; B) UELtrap-EA; C) CRtrap-E; D) CRtrap-EA”.

**In the text the other requested information was added containing details of the trap structure. The new wording was**: “Four types of traps adapted from the original [44, 45] were tested (Fig 2): i) UELtrap-E (standard trap) for egg capture (black rounded plastic vase measuring 12 cm length x 11 cm diameter, with a capacity of 750 mL) (Fig 2A), ii) UELtrap-EA for capture of eggs and adults (12 x 11 cm black rounded plastic vase that is 750 ml in volume, with side openings and a lid with a tulle for ventilation on the top and contains a funnel coated with commercial entomological glue Colly^®^) (Fig 2B), (iii) CRtrap-E for egg capture (clear circular plastic container measuring 8 cm length x 9 cm diameter, with capacity of 500 mL, contains a black cone with a rough part to facilitate oviposition and egg adhesion) (Fig 2C) and (iv) CRtrap-EA for capturing eggs and adults (8 x 9 cm clear circular plastic container, containing a roughened black outer cone and a lid associated with a funnel coated with commercial entomological glue Colly^®^) (Fig 2D).

Fig 2. Traps for capture of *Aedes* eggs and adults under field conditions: A) UELtrap-E; B) UELtrap-EA; C) CRtrap-E; D) CRtrap-EA.

The UELtrap-E and UELtrap-EA traps have a Duratree Eucatex^®^ reed that measures 13 cm length x 3 cm width, positioned vertically with the rough surface facing upwards to facilitate oviposition and egg adhesion. In contrast, the CRtrap-E and CRtrap-EA have 8 cm length x 1 cm width brown plastic reeds with both smooth and rough surfaces that are placed upright with the rough part facing the outside of the opening from the container.

The traps were optimized for the collection of pregnant females of *Aedes aegypti* and *Aedes albopictus* for the purpose of simplicity and low cost of around US $ 2.5 per unit.

- Collection eggs and adults of *Aedes* under field conditions: Please inform if the traps were and how were cleaned every 7 days? Were attractant solutions replaced every 7 days? Were attractant solutions transported to entomological laboratory to identify immature *Aedes* (larvae and pupae)? How was the material transported to the laboratory? How many people were needed to monitor the traps (field and laboratory).

**The authors updated the text to meet the requested demands, thus including more details in the procedure for collecting and reading eggs and adults. Also included in the new text are federal and institutional collection authorizations: “**Each trap was given 250 mL of water without chlorine and 50 mL of solution containing *M. maximus* (0.11256%) [37] and the biological product Vectobac^®^ WG (0.00083%) (Active ingredient: *B. thuringiensis* *israelensis*), strain AM65-52, 37.4% w/w; Lot No.: 267-853-PG; Date of manufacture: July 2016; Valent BioSciences Corporation - VBC). The attractant solution (50 mL) used in the traps was obtained from a 50 mg/L dilution of the biological product in 5 L of grass infusion (0.0050% and 0.6754%, respectively).

The reeds from UELtrap-E and UELtrap-EA were replaced every seven days and sent in plastic basins containing absorbent paper to the Medical Entomology Laboratory of Londrina State University, and to the Biological Control and Biotechnology of Malaria and Dengue Laboratory at INPA, where the eggs were quantified with the aid of a 50x stereoscope microscope, after drying the reeds at room temperature. The attractive solution of the traps was discarded and replaced every seven days, while the traps were not exchanged.

The eggs present on the plastic reeds and inside CRtrap-E and CRtrap-EA were quantified *in situ* with the aid of a manual magnifying glass (10x) and double-sided tape for egg removal since the reeds and traps were not replaced. On the other hand, the attractive solution was discarded and replaced every seven days.

Adults collected with UELtrap-EA and CRtrap-EA were removed with the aid of entomological forceps and stored in glass bottles containing absorbent paper to preserve the integrity of the characteristics. Mosquitoes were counted and identified at the species level using external morphological characters with the aid of stereomicroscopy and the identification keys proposed by [1, 2, 47]. For laboratory and field work, three team members were needed.

Collections in the different study sites were carried out using the Sisbio / Ibama authorization: 23093 and 65287 licenses. In UTFPR Process nº 23064.025800 / 2018-21 and in the National Institute of Research of the Amazon the authorization is under number PRJ06.173.”

The results are clear and well organized
with graphics and tables. However, there are some important issues:

The proposal of the manuscript is to evaluate the effectiveness of 4 traps for the collection and capture of urban mosquitoes of the genus *Aedes*. The analyzes were carried out for the aggregated data over time, however the authors could inform the collects points with high and low number of the eggs and adults (male and female) collected per species. What factors could be associated with this finding, even knowing that it is a short period of time (in the discussion).

- Inform how many males and females were caught by adult mosquito species and by trap.

**The authors maintained the graphs and tables that complement each other in the various assessments in the different regions and traps used. The text contained information on how many adult mosquitoes were caught, but there was little emphasis. The authors allocated the text for greater visibility. The new wording had separate paragraphs within the analysis of the result in the north of Paraná and also in the north. The results are described within each sub-item “Total eggs and adults ……. As below:**

The UELtrap-EA traps captured 17 female specimens; one *A. albopictus*, ten *A. aegypti* and six *Culex quinquefasciatus* Say, 1823. Regarding the percentage of adults collected from each species, 6%, 59% and 35% were found for the species *A. albopictus*, *A. aegypti* and *C. quinquefasciatus*, respectively. According to equations 1 and 2, this trap model had a female capture ratio of *Aedes* spp. Of 29.50%. This indicated that approximately 29.50% of incoming females were caught. On the other hand, Crtrap-EA captured only one *C. quinquefasciatus* female*.*

In the UELtrap-EA traps, 25 female specimens were obtained: 23 *A. albopictus*, one *Limatus* spp. and one *Limatus* *durhamii* Theobald, 1901, representing percentages of 92%, 4% and 4%, respectively. Based on equations 1 and 2, these traps presented a female *Aedes* spp. capture ratio of 53.51%. This indicated that approximately 53.51% of the females who entered the traps were caught. On the other hand, in the CRtrap-EA traps, only one *Aedes* spp. female was captured. The capture ratio of *Aedes* spp. female for this trap was 9.80%. Therefore, approximately 9.80% of the females that entered were captured.

**Reviewer #1**: Aedes aegypti and Ae. albopictus are the most well know Culicidae species. However, according to the literature, the difficulties of controlling these species in large and medium-sized cities of Brazil are numerous. Considering their easy proliferation and the limitations to reduce their infestation rates (generated by the complexity of current urban life), to multiply the investigations with this approach is relevant. However, this study has some important issues to be addressed, and the main concerns are its short sampling period and the capture concentration. Thus, I recommend the publication of this study under major revisions.

**The methodology of the article was based on the technical literature with n sample that could validate the results obtained. Statistically, there is a clear difference between the traps according to the proposed objective. The results were identical in the south and north of the country, which was another initial proposal, to ascertain their behavior in different areas in the country with different climate and infestations of these mosquitoes also different.**

**The work had theoretical foundations to design the sample n in literature that has similarities to replicas and traps in the study areas with the one developed here. We used 4 different traps with 05 aerial traps in 05 termporal replicates so we quote below some works that provided us with information to draw the sample n:**

**Field evaluation of a lethal ovitrap against dengue vectors in Brazil. M. J. Perich A. Kardec I. A. Braga I. F. Portal R. Burge B. C. Zeichner W. A. Brogdon R. A. Wirtz**

**First published: 19 June 2003** [**https://doi.org/10.1046/j.1365-2915.2003.00427.x**](https://doi.org/10.1046/j.1365-2915.2003.00427.x)

**Comparison Between Larval Survey Index and Positive Ovitrap Index in the Evaluation of Populations of Aedes (Stegomyia) aegypti (Linnaeus, 1762) North of Paraná, Brazil. Kauani Larissa Campana Nascimento, João Fernando Marques da Silva, João Antonio Cyrino Zequi, ...**

**First Published January 6, 2020 Research Article Find in PubMed**

**https://doi.org/10.1177/1178630219886570**

### Oviposition of Aedes aegypti Linnaeus, 1762 and Aedes albopictus Skuse, 1894 (Diptera: Culicidae) under laboratory and field conditions using ovitraps associated to different control agents, Manaus, Amazonas, Brazil. William Ribeiro da Silvaa Joelma Soares-da-Silvab Francisco Augusto da Silva Ferreiraa Iléa Brandão Rodriguesa Wanderli Pedro Tadei. Rev. Bras. entomol. vol.62 no.4 São Paulo Oct./Dec. 2018 <http://dx.doi.org/10.1016/j.rbe.2018.08.001>

### Identification of the best ovitrap installation sites for gravid *Aedes (Stegomyia) aegypti* in residences in Mirassol, state of São Paulo, Brazil Mem. Inst. Oswaldo Cruz vol.100 no.4 Rio de Janeiro July 2005

#### http://dx.doi.org/10.1590/S0074-02762005000400001

**Margareth Regina Dibo^I,^**[**^1^**](https://www.scielo.br/scielo.php?script=sci_arttext&pid=S0074-02762005000400001#end)**; Francisco Chiaravalloti-Neto^II^; Marcos Battigaglia^I^; Adriano Mondini^II^; Eliane A Favaro^II^; Angelita AC Barbosa^II^; Carmen M Glasser^I^**

Reviewer #2: The authors informed that the objectives of the study were "to evaluate the efficiency of different traps for oviposition and capture of A. aegypti and A. albopictus adults in field conditions in South and North Regions of Brazil to validate new tools that can be effective and economical for vector monitoring", but they also concluded that the traps could be useful for reducing eggs and adults of Aedes spp. I agree that the traps could be useful to measure the entomological indicators (and that the authors used suitable methods to evaluate the traps from this point of view), but not as a way to reduce the number of mosquitoes. Furthermore, this was not the objective of the study. For testing this hypothesis, the study would have a different design.
**The authors are in full agreement with the reviewer, and have eliminated the mention of control throughout the text, as it was not really the objective of the work and the methodology is also not aligned to verify control. The old and new version of the article makes this aspect clear.**

Reviewer #4: - The objectives of the study are not clearly articulated with a clear testable hypothesis stated.
- As the traps are adaptations, I missed the specifications and photos to better understand the model, which lack details.

**The authors included more details about the traps and also a board with a photo of the traps as indicated in the new text as Fig. 2. The authors describe the text just above with their justifications for the reviewer 3.**

- The sample size was not sufficient to ensure adequate power to address the hypothesis being tested. The installation time of the traps in the field (5 weeks) in a single season does not allow the visualization of their efficacy in different climatic conditions. As a result, important issues of seasonality and trap performance are lost over the course of a year.

**In the south of the country, as mentioned in the new text, there is a description of the climate of each studied location with its variations as follows:**

The climate of Londrina is humid subtropical, with an average annual temperature of around 22 °C, relative humidity of around 70%, having hot summer and rain in all seasons (annual average between 1400 and 1800 mm) [42]. The study area is situated in the urban perimeter of Londrina and is daily attended by a high number of students and employees, constituting an important area for investigation and monitoring of mosquito vectors of pathogens.

In Manaus, the climate is humid equatorial with an average annual temperature of 26 °C, and a relative humidity of around 80%, with an annual rainfall of around 2,300 mm. The region has two well-defined seasons: rainy (December to June) and dry (July to November), based on rainfall and river levels [43].

The study site is a very wooded urban environment with native forest, which favors *A. albopictus* [2]*.* As in Londrina, the *Campus* are full of students and employees, being part of the local community.

#### With the information in hand, the traps were installed for 5 weeks at times favorable to the mosquito in the different regions, because with a drop in temperature there is a clear drop in infestation in the south of the country, but the trap, mainly the standard, continues to generate indices with low values. In the north of the country the infestation is practically the same during the year. As the objective was to verify the effectiveness of the trap in two different regions, time constancy was not addressed, but many scientific articles bring this information with the standard trap used in this experiment, such as: Nascimento et al., 2020. Comparison Between Larval Survey Index and Positive Ovitrap Index in the Evaluation of Populations of Aedes (Stegomyia) aegypti (Linnaeus, 1762) North of Paraná, Brazil. <https://doi.org/10.1177/1178630219886570> and Morato et al. 2005. Infestation of Aedes aegypti estimated by oviposition traps in Brazil. <http://dx.doi.org/10.1590/S0034-89102005000400006>

**The sample size used was based on technical literature with no sample that could validate the results obtained. Statistically, there is a clear difference between the traps according to the proposed objective. The results were identical in the south and north of the country, which was another initial proposal, to ascertain their behavior in different areas in the country with different climate and infestations of these mosquitoes also different.**

**The work had theoretical foundations to design the sample n in literature that has similarities to replicas and traps in the study areas with the one developed here. We used 4 different traps with 05 aerial traps in 05 termporal replicates so we quote below some works that provided us with information to draw the sample n:Field evaluation of a lethal ovitrap against dengue vectors in Brazil. M. J. Perich A. Kardec I. A. Braga I. F. Portal R. Burge B. C. Zeichner W. A. Brogdon R. A. Wirtz**

**First published: 19 June 2003** [**https://doi.org/10.1046/j.1365-2915.2003.00427.x**](https://doi.org/10.1046/j.1365-2915.2003.00427.x)

**Comparison Between Larval Survey Index and Positive Ovitrap Index in the Evaluation of Populations of Aedes (Stegomyia) aegypti (Linnaeus, 1762) North of Paraná, Brazil. Kauani Larissa Campana Nascimento, João Fernando Marques da Silva, João Antonio Cyrino Zequi, ...**

**First Published January 6, 2020 Research Article Find in PubMed**

**https://doi.org/10.1177/1178630219886570**

### Oviposition of Aedes aegypti Linnaeus, 1762 and Aedes albopictus Skuse, 1894 (Diptera: Culicidae) under laboratory and field conditions using ovitraps associated to different control agents, Manaus, Amazonas, Brazil. William Ribeiro da Silvaa Joelma Soares-da-Silvab Francisco Augusto da Silva Ferreiraa Iléa Brandão Rodriguesa Wanderli Pedro Tadei. Rev. Bras. entomol. vol.62 no.4 São Paulo Oct./Dec. 2018 <http://dx.doi.org/10.1016/j.rbe.2018.08.001>

### Identification of the best ovitrap installation sites for gravid *Aedes (Stegomyia) aegypti* in residences in Mirassol, state of São Paulo, Brazil Mem. Inst. Oswaldo Cruz vol.100 no.4 Rio de Janeiro July 2005

#### http://dx.doi.org/10.1590/S0074-02762005000400001

**Margareth Regina Dibo^I,^**[**^1^**](https://www.scielo.br/scielo.php?script=sci_arttext&pid=S0074-02762005000400001#end)**; Francisco Chiaravalloti-Neto^II^; Marcos Battigaglia^I^; Adriano Mondini^II^; Eliane A Favaro^II^; Angelita AC Barbosa^II^; Carmen M Glasser^I^**

- The study design was not appropriate to address the stated objectives. The traps remained in the same position over the five weeks, which can generate an installation bias. Ideally, they would have passed through a Latin square experimental design.

**As shown in Figure 1, the traps were placed in the north and south of the country at a distance of 25 meters each using 05 traps of each model arranged interspersed with each other and with 05 replicates of collections in different weeks by traps. Considering the biology of Aedes aegypti and Aedes albopictus to Brazil, where in a flight radius of up to 500 meters the populations are structurally very different. In press article: ““ Genetic study in Aedes (Stegomyia) aegypti (Linnaeus, 1762) from Londrina (Paraná State, Brazil): an approach to population structure and pyrethroid resistance ”Barbosa et al. Revista Brasileira de Entomologia. It is unlikely that the traps may undergo changes in terms of infestation due to the fact that they are not rotated, since the environments of each installed block are similar in physical structure and climatic conditions (see description of the reformulated study area with more climate details more details of vegetation and structures of the area (response included and in the final text due to what was requested by the reviewer 3 as well).**

- The authors cited only a collection license and did not address ethical issues or even limitations of the study.
**The authors included approved licenses for the study. Federal and local licenses for each institution. This authorization already includes all the necessary requirements for the development of the research, including ethics. The new paragraph was:**

“Collections in the different study sites were carried out using the Sisbio / Ibama authorization: 23093 and 65287 licenses. In UTFPR Process nº 23064.025800 / 2018-21 and in the National Institute of Research of the Amazon the authorization is under number PRJ06.173.”

--------------------

**Results**

Reviewer #1: The main concerns are the shortcomings of the study design and limited results. I recommend that the authors make a more accurate description, in addition to referencing an image of the traps, to facilitate the understanding of their functionality. It is important to emphasize that it is not a new trap but characterizes a new adaptation of existing traps, the UELtrap-E and UELtrap-EA, which capture A. aegypti and A. albopictus eggs, respectively.

**The authors took care to mention that the trap is an adaptation. A board was also included (Fig.2) with the details of the trap and descriptive in the text. The new text reads:** “Trap characteristics

Four types of traps adapted from the original [44, 45] were tested (Fig 2): i) UELtrap-E (standard trap) for egg capture (black rounded plastic vase measuring 12 cm length x 11 cm diameter, with a capacity of 750 mL) (Fig 2A), ii) UELtrap-EA for capture of eggs and adults (12 x 11 cm black rounded plastic vase that is 750 ml in volume, with side openings and a lid with a tulle for ventilation on the top and contains a funnel coated with commercial entomological glue Colly®) (Fig 2B), (iii) CRtrap-E for egg capture (clear circular plastic container measuring 8 cm length x 9 cm diameter, with capacity of 500 mL, contains a black cone with a rough part to facilitate oviposition and egg adhesion) (Fig 2C) and (iv) CRtrap-EA for capturing eggs and adults (8 x 9 cm clear circular plastic container, containing a roughened black outer cone and a lid associated with a funnel coated with commercial entomological glue Colly®) (Fig 2D).

Reviewer #2: As presented above, the data analysis the authors did was suitable for the evaluation of the traps of the point of view to monitoring the infestation levels of Aedes, but not to conclude that the traps could be useful to control mosquitoes. The authors prioritized, in the Results, the comparison of the traps two by two (UELtrap-E with CRtrap-E and UELtrap-EA with CRtrap-EA). But, taking into account the study design (the four traps put togheter), they colud present the results comparing the four traps. Also, the performance of UELtrap-E was higher than UELtrap-EA in Manaus (a differente result of they found in Londrina), but the authors did not give sufficient attention to this issue in the presentation of results.

**The authors agree with the reviewer, and as far as possible, we express the results of the different traps with statistics in the form of tables, graphs and descriptors in the text, similar to the collection of adults. As the infestation in Manaus is much larger than in the south of the country and is mainly related to Aedes albopictus this difference between UELtrap-E was higher than UELtrap-EA in Manaus was expected, since it is the traditional trap and the most sensitive according to the note Fiocruz technique: http://www.fiocruz.br/ioc/media/nota_tecnica_ioc_3.pdf This result was possibly not found in the south of the country possibly due to a minor infestation. Even UELtrap-E was higher than UELtrap-EA (see table 4) do not differ statistically, so the authors found it not necessary to discuss this numerical difference, since the trap is an adaptation of the standard ovitrap with the advantage of collecting the adult.**

Reviewer #3: The results are clear and well organized
with graphics and tables. However there are some important issues:
The proposal of the manuscript is to evaluate the effectiveness of 4 traps for the collection and capture of urban mosquitoes of the genus Aedes. The analyzes were carried out for the aggregated data over time, however the authors could inform the collects points with high and low number of the eggs and adults (male and female) collected per species. What factors could be associated with this finding, even knowing that it is a short period of time (in the discussion).
- Inform how many males and females were caught by adult mosquito species and by trap.

**The traps were installed at times suitable for mosquitoes in the southern and northern regions, which is practically all year round. The authors placed more emphasis on the discussion and also on the introduction better characterizing the climate and also the local vegetation of each location and the meeting of the different infestations in the traps. They also mentioned the adults captured in each south and north sub-area to facilitate the explanation of the results and discussion. The text containing the adult information was:**

“The UELtrap-EA traps captured 17 female specimens; one *A. albopictus*, ten *A. aegypti* and six *Culex quinquefasciatus* Say, 1823. Regarding the percentage of adults collected from each species, 6%, 59% and 35% were found for the species *A. albopictus*, *A. aegypti* and *C. quinquefasciatus*, respectively. According to equations 1 and 2, this trap model had a female capture ratio of *Aedes* spp. Of 29.50%. This indicated that approximately 29.50% of incoming females were caught. On the other hand, Crtrap-EA captured only one *C. quinquefasciatus female.*

In the UELtrap-EA traps, 25 female specimens were obtained: 23 *A. albopictus*, one *Limatus* spp. and one *Limatus* *durhamii* Theobald, 1901, representing percentages of 92%, 4% and 4%, respectively. Based on equations 1 and 2, these traps presented a female *Aedes* spp. capture ratio of 53.51%. This indicated that approximately 53.51% of the females who entered the traps were caught. On the other hand, in the CRtrap-EA traps, only one *Aedes* spp. female was captured. The capture ratio of *Aedes* spp. female for this trap was 9.80%. Therefore, approximately 9.80% of the females that entered were captured”.

Reviewer #4: -
- The figures (Tables, Images) are not of sufficient quality for clarity. Photos or schematics were missing for better understanding the traps. Figures 1 and 2 lack subtitles, study period and standard deviation.

**The authors included a board with pictures of the traps and also details in the text written on them. The figures had their resolution improved as can be seen in the annexes. All subtitles are available in the text and in context.**

- The first paragraph on the total of eggs and adults collected is actually the discussion of the data and not the presentation of the results.

**The first paragraph was revised and emphasized the comparative results as described below:**

“Considering the traps used exclusively for egg capture, it was observed that in the UELtrap-E traps more eggs were obtained than in the CRtrap-E traps (Fig 3). This result was corroborated when analyzing the average number of eggs obtained for both, since the first obtained an average (341) 18 times higher than the value (19) obtained in the second trap, thus presenting a statistically significant difference between the respective values (p = 0.0090) (Table 1).”

- CRTrap's performance was better in Manaus than in Londrina, which may be related to the difference in the collection periods and perhaps the installation locations. Since the Latin square model was not considered, one cannot answer about the installation bias.

**As contextualized in the introduction and results presented, mosquito infestation is much higher in the north compared to the south, which would naturally occur greater collection of eggs in the same trap model. This occurred with all the traps as shown in the graphs of Figures 3 and 4.**

- In discussion section, line 369 : "The light coloration of the traps CRtrap-E and CRtrap-EA may also have influenced egg laying. According to [2] females ... " they put just the number as a citation, that´s correct? The same im line 400 and other places.
**The text was changed and more literature was added in addition to 2**.

**The new text was:** “The light coloration of the traps CRtrap-E and CRtrap-EA may also have influenced egg laying. According to [2, 52] females of the genus *Aedes* prefer darker places for oviposition. This fact explains the preference of the females in choosing black traps during egg laying. Therefore, the average numbers of eggs obtained in the UELtrap-E and UELtrap-EA traps and the high values of OPI and EDI observed in both regions indicate that these two trap models (dark color and rough surface) were more inviting to *Aedes* spp. females.”

--------------------

**Conclusions**

Reviewer #1: The manuscript is relevant to public health. There are limits to the analysis, considering the insufficient quantity of traps used at the different collection points to conduct this research.

Reviewer #2: Taking into account the obtained results, the authors should conclude only about the use of traps to monitor Aedes infestation, but nothing about the use of traps for vector control. The study design used by the authors was not suitable to evaluate this issue.

Reviewer #3: (No Response)

Reviewer #4: - The conclusions was not supported by the data presented. The original ovitrampa also has high sensitivity and specificity and no comparison was made with the standard methodology to compare the results. Thus, the recommendation to use the adapted model as a most effective, practical and economical way to
monitor A. aegypti and A. albopictus on a local scale is not justified.

- The limitations of analysis was not clearly described

- Public health relevance was addressed

--------------------

**The authors took great care in this new wording to include only monitoring with traps, not to mention control, since it is not the focus of the objective and the methodology also did not have this purpose. The results presented with statistics make it clear that it is more advantageous to use a trap that can capture adults in percentages with more than 50% cases of greater infestation, for example in the north of the country, than the standard trap that does not capture adults. This fact is confirmed in the article by means of statistics, graphs and tables as also described in the other questions that the authors attended to in full and that greatly improved the article. The emphasis is that the adapted trap can contribute to monitoring plans in government actions at low cost. The new wording of the conclusion then became:**

These traps do not inconvenience those in the installation areas or to the health workers who should be charged with monitoring the traps since they do not need to be installed indoors but rather in open areas with a large flow of people, such as outside of universities, institutes and industrial buildings as well as in peridomiciles. These traps are an operationally viable and noninvasive method and may become the most effective, practical and economical way to monitor *A. aegypti* and *A. albopictus* on a local scale, provided that the traps are monitored weekly by technical staff.

The entire process can be reconciled with official government strategies for more accurate vector monitoring that can support actions with the population for local surveys and greater efficiency in vector control when necessary.
